# Lipocalin 2 is present in the EAE brain and is modulated by natalizumab

**DOI:** 10.3389/fncel.2012.00033

**Published:** 2012-08-09

**Authors:** Fernanda Marques, Sandro D. Mesquita, João C. Sousa, Giovanni Coppola, Fuying Gao, Daniel H. Geschwind, Sandra Columba-Cabezas, Francesca Aloisi, Matilda Degn, João J. Cerqueira, Nuno Sousa, Margarida Correia-Neves, Joana A. Palha

**Affiliations:** ^1^School of Health Sciences, Life and Health Sciences Research Institute (ICVS), University of MinhoBraga, Portugal; ^2^ICVS/3B's - PT Government Associate LaboratoryBraga/Guimarães, Portugal; ^3^Program in Neurogenetics, Department of Neurology, David Geffen School of Medicine-University of California, Los Angeles, Los AngelesCA, USA; ^4^Department of Cell Biology and Neuroscience, Istituto Superiore di SanitàRome, Italy; ^5^Department of Neurology, Glostrup HospitalGlostrup, Denmark; ^6^Clinical Academic CenterBraga, Portugal

**Keywords:** multiple sclerosis, lipocalin 2, experimental autoimmune encephalomyelitis, astrocytes, cerebrospinal fluid, natalizumab

## Abstract

Multiple sclerosis (MS) is a demyelinating disease that causes major neurological disability in young adults. A definitive diagnosis at the time of the first episode is still lacking, but since early treatment leads to better prognosis, the search for early biomarkers is needed. Here we characterized the transcriptome of the choroid plexus (CP), which is part of the blood-brain barriers (BBBs) and the major site of cerebrospinal fluid production, in the experimental autoimmune encephalomyelitis (EAE) mouse model of MS. In addition, cerebrospinal fluid samples from two cohorts of patients with MS and with optic neuritis (ON) were analyzed to confirm the clinical relevance of the findings. Genes encoding for adhesion molecules, chemokines and cytokines displayed the most altered expression, supporting the role of CP as a site of immune-brain interaction in MS. The gene encoding for lipocalin 2 was the most up-regulated; notably, the cerebrospinal fluid lipocalin 2 levels coincided with the active phases of the disease. Immunostaining revealed that neutrophils infiltrating the CP were the source of the increased lipocalin 2 expression in this structure. However, within the brain, lipocalin 2 was also detected in astrocytes, particularly in regions typically affected in patients with MS. The increase of lipocalin 2 in the cerebrospinal fluid and in astrocytes was reverted by natalizumab treatment. Most importantly, the results obtained in the murine model were translatable into humans since patients from two different cohorts presented increased cerebrospinal fluid lipocalin 2 levels. The findings support lipocalin 2 as a valuable molecule for the diagnostic/monitoring panel of MS.

## Introduction

Multiple sclerosis (MS) is a chronic progressive inflammatory disease of the central nervous system (CNS) characterized by an immune response against the myelin sheath of CNS axons. A notable aspect of the disease is the migration of inflammatory cells through the brain barriers towards the CNS. These cells, together with activated astrocytes and microglia, are likely to induce the demyelination and neurodegeneration observed in the MS lesions, ultimately resulting in the loss of motor functions (Brown and Sawchenko, 2007; Vercellino et al., 2009). As such, current clinical therapies are greatly directed at inhibiting lymphocyte trafficking to the CNS (Hemmer et al., 2006).

The blood-brain barrier (BBB), formed by the endothelial cells of brain capillaries, and the blood-cerebrospinal fluid (CSF) barrier, formed by the choroid plexus (CP) epithelial cells, are the main interfaces between the CNS and the periphery. In MS, it appears that the first wave of CNS-infiltrating T cells, specifically T helper 17 (Th17), enter through the CP; this primary event triggers the entry of a second T cell wave that migrates in large numbers through the BBB into the CNS (Reboldi et al., 2009). In addition, the brain barriers are also of primary interest given their secretome, which, via the CSF (mostly produced by the CP) or by direct action on the brain parenchyma, may modulate brain homeostasis (Zlokovic, 2008; Marques et al., 2009a). Findings attesting the relevance of the CSF-CP in MS also include the observation that most MS lesions localize in periventricular areas and that the CSF composition may participate in disease development, a contribution that remains poorly understood. The CSF composition is reflective of brain metabolism, thus, CSF analysis is a useful diagnostic tool for neurological diseases (Tumani et al., 2009). The search for biomarkers that correlate with MS disease progression and activity is particularly needed given that early treatment can lead to a more favorable prognosis (Comi et al., 2000). To further understand whether the brain barrier at the CP contributes to MS, and to search for novel biomarkers, we characterized here the CP transcriptome in the onset, remission and relapse phases of the murine experimental autoimmune encephalomyelitis (EAE) model of MS. To verify if the findings were of relevance to the human disease, we also assessed the CSF composition from two distinct human cohorts of MS.

## Methods

### Animals and induction of EAE

All experiments were conducted using 6–8 weeks of age adult female SJL mice (Charles River, Barcelona, Spain) in accordance with the Portuguese national authority for animal experimentation, Direção Geral de Veterinária (ID: DGV9457). Animals were kept and handled in accordance with the guidelines for the care and handling of laboratory animals in the Directive 2010/63/EU of the European Parliament and of the Council. The animals were housed and maintained in a controlled environment at 22–24°C and 55% humidity, on 12 h light/dark cycles and fed with regular rodent's chow and tap water *ad libitum*. Animals were handled for 1 week prior to the beginning of the experiment, in order to reduce the stress induced by the injections.

For the induction of the relapsing-remitting EAE model, female SJL mice were subcutaneously injected in the hind flanks, with 0.2 mg of the encephalitogenic proteolipid protein (PLP) 139–151 peptide (HSLGKWLGHPDKF) (Primm, Milan, Italy), in Complete Freund's Adjuvant (CFA; Difco Laboratories, Detroit, MI, USA), on days 0 and 7. Each mouse was also intraperitoneally injected with 0.2 μg of pertussis toxin (Sigma, St. Louis, MO, USA) on days 0, 1, 7, and 8. Control mice were injected with phosphate buffer saline (PBS) in CFA and pertussis toxin, according to the same schedule (Columba-Cabezas et al., 2006). All mice were weighed and examined daily for the clinical signs of EAE, which was scored according to the following scale: grade 0, no abnormality; grade 1, reduced tail tonus or slight clumsy gait; grade 2, tail atony, moderately clumsy gait, impaired righting ability, or any combination of these signs; grade 3, additional hind limb weakness; grade 4, hind limb paralysis and fore limb weakness; grade 5, tetraplegia or moribund state. Mice developed an acute form of EAE with a peak between days 12 and 14 (grades 1–4), which was termed onset. A phase of complete remission took place between days 19 and 22, with some mice undergoing a relapse (grades 1 and 2) after a variable period of time (27–31 days).

### Human samples

For the human studies, CSF samples were obtained according to standardized protocols and refer to patients admitted for lumbar puncture in the Hospital de Braga, Portugal, and in the Glostrup Hospital, Denmark, between February 2009 and September 2011. Patients were referred to the Neuroimmunology Clinic, Hospital de Braga, Portugal, or the Clinic of Optic Neuritis, Glostrup Hospital, Denmark and underwent diagnostic program including magnetic resonance imaging (MRI) scan and lumbar puncture. Lumbar puncture was performed within 30 days of symptom debut, thus in the active phase of disease. The groups included in this study were controls, optic neuritis (ON) patients and patients with clinically definite relapsing remitting MS. Controls from Braga were individuals, of similar age and gender as cases that were referred to the neurology department because of neurological complaints, who were found to have no CNS disorder and normal CSF examination; Glostrup controls were age and gender matched, with no genetical disposition to autoimmune disease and had a normal neurological examination. Selected ON patients, all from Glostrup, were in the lower end of the risk to develop MS, one ON patient had a single white matter lesion and one had pathological CSF, the remaining had no pathological finding in CSF and in the MRI. All MS patients, from both Braga and Glostrup, were diagnosed according to the McDonald 2005 criteria (Polman et al., 2005); additionally, all except three had pathological CSF with oligoclonal bands (OCB) or increased IgG-index. The studies with human CSF were approved by the Danish ethical committee and the ethical committee of Hospital de Braga and all patients and controls signed an informed consent.

### Natalizumab treatment

In the natalizumab treatment protocol, EAE was induced and after appearance of the first clinical signs animals were injected intraperitoneally with 5 mg/Kg of anti-VLA-4 antibody (natalizumab) (Biogen Idec, Boston, MA, USA) or in the case of control mice with rat IgG (Sigma). To better represent clinical use, treatment injections were performed during the active phase (1, 3, and 5 days after the appearance of the symptoms) (i.e., reverting symptoms) (Theien et al., 2001). Animals were sacrificed at day 14 (that corresponds to the onset phase for animals not treated with natalizumab).

### Tissue and CSF collection

Mice were sacrificed during the first peak of disease (onset), during the remission phase and in the relapse phase. Mice from the control group were sacrificed on day 14, along with mice from the onset group. Animals were anesthetized with ketamine hydrochloride (150 mg/Kg) plus medetomidine (0.3 mg/Kg), CSF samples were collected from the cisterna magna, and animals were transcardially perfused with cold saline. CSF pooled samples were checked for blood contamination and stored at –80°C. After perfusion the CP's were rapidly removed from each mouse ventricle under conventional light microscopy (SZX7, Olympus, Hamburg, Germany), frozen in dry ice and stored at –80°C.

For the expression studies, two sets of animals from each experimental group (control, onset, remission, and relapse) were prepared: one for the microarray analysis containing 3 separate pooled CP samples for onset and remission phase and 2-pooled CP for the relapse phase (from three animals each) and another to the quantitative Real Time-Polymerase Chain Reaction (qRT-PCR) containing at least from 5 pools of CP (from three animals each).

### Microarray experimental design and data analysis

Total RNA was isolated with Trizol (Invitrogen, Carlsbad, CA, USA) following manufacturer's instructions. After quality assessment using the Agilent Bioanalyzer (Agilent Technologies, CA, USA), 100 ng of total RNA were amplified and labeled with Illumina TotalPrep RNA Amplification Kit (Illumina Inc., San Diego, CA, USA). The labeled cRNA was then hybridized using the recommended protocol in a total of two Illumina Whole-genome Mouseref-8 expression Beadchips (Illumina Inc., San Diego, CA, USA). This mouse beadchip contains eight arrays, each comprising a total of 24,000 well-annotated RefSeq transcripts. After scanning, raw data from BeadStudio software (Illumina Inc., San Diego, CA, USA) was read into R/Bioconductor and normalized using quantile normalization. A linear model was applied to the normalized data using Limma package in R/Bioconductor. A contrast analysis was applied and differentially expressed genes were selected using a Bayesian approach with a false discovery rate of 5%. All data is Minimum Information About a Microarray Experiment (MIAME)-compliant and the raw data has been deposited in the GEO database. The differentially expressed genes were categorized using Gene Ontology from Biomart (http://www.biomart.org/) or Ingenuity tools (Redwood City, CA, USA). Enrichment analysis was performed using the DAVID (http://david.niaid.nih.gov/david/ease.htm) and the Ingenuity softwares.

### Gene expression measurements by qRT-PCR

Total RNA was isolated from the CP as described before. An amount of 500 ng of RNA from each pool was amplified using a SuperScript RNA Amplification System (Invitrogen) according to the manufacturer's instructions. After amplification, RNA was reverse transcribed into first strand cDNA using random hexamers of the superscript first-strand synthesis system for RT-PCR (Invitrogen). Primers used to measure the expression levels of selected mRNA transcripts by qRT-PCR were designed using the Primer3 software, on the basis of the respective GenBank sequences. All accession numbers and primer sequences are available on request. The reference gene for hypoxanthine guanine phosphoribosyl transferase (*Hprt*) (accession number from GenBank: NM_013556) was used as an internal standard for the normalization of the expression of selected transcripts. qRT-PCR was performed on a CFX 96TM real time system instrument (Bio-Rad Laboratories, Hercules, CA, USA), with the QuantiTect SYBR Green RT-PCR reagent kit (Qiagen, Hamburg, Germany) according to the manufacturer's instructions, using equal amounts of RNA from each sample. Product fluorescence was detected at the end of the elongation cycle. All melting curves exhibited a single sharp peak at the expected temperature.

### Immunohistochemistry and immunofluorescence analyses

Animals were transcardially perfused with saline, under anesthesia, at the various time points mentioned before. After perfusion, brains were removed from the skull, immediately embedded in Tissue-Tek optimal cutting temperature compound and kept frozen at –20°C. Brains were sectioned in serial 20 μm cryostat coronal sections, which were then fixed with 4% PFA in PBS. After antigen retrieval with citrate buffer 10 mM, the sections were probed with the primary antibody, anti-mouse lipocalin-2/neutrophil gelatinase-associated lipocalin (LCN2/NGAL) (1:400; R&D Systems, MN, USA), diluted in PBS 0.3% Triton X-100 (PBS-T), and 0.4% bovine serum albumin (Sigma). Afterwards the sections, were incubated with biotinylated anti-goat secondary antibody and then with streptavidin peroxidase conjugate (ABC kit; Sigma). Reaction was developed with 3,3′-diaminobenzidine tetrahydrochloride hydrate (DAB; Sigma) and sections stained with hematoxylin. Alternatively, for immunofluorescent staining, sections were incubated with goat anti-mouse LCN2/NGAL in conjunction with: rabbit anti-glial fibrillary acidic protein (anti-GFAP) (1:200; Dako, Glostrup, Denmark) to label astrocytes, rat anti-mouse Ly-6G to label neutrophils (1:200; clone 1A8, BD Biosciences, Franklin Lakes, NJ, USA), mouse anti-CNPase to label oligodendrocytes [1:100; Chemicon (Millipore), Billerica, MA, USA], mouse anti-NeuN (1:100; Millipore, Billerica, MA, USA) to label neurons and rabbit anti-Iba1 (1:200; Wako chemicals, Richmond, VA, USA) to label microglia. The appropriate secondary fluorescent antibodies diluted 1:500 in PBS-T were used: anti-goat Alexa 488, anti-rabbit and anti-mouse Alexa 594, anti-rat Alexa 547 (all from Invitrogen). For each analysis, sections from 3–5 different animals per group were used. The cell nucleus was stained using 4′, 6-diamidino-2-phenylindole (DAPI). Samples were analyzed using optical (BX61; Olympus) or confocal (FV1000; Olympus) microscopes.

### LCN2 quantification in mice and human CSF

LCN2 protein determination in the CSF was done by a direct enzyme-linked immunosorbent assay, using 2 μl of CSF for mice and 5 μ l for humans. LCN2 was detected using the goat anti-mouse LCN2/NGAL or anti-human LCN2/NGAL (both from R&D Systems), at 1:300 dilution, followed by a secondary peroxidase-conjugated donkey anti-goat antibody (Santa Cruz Biotechnology, Santa Cruz, CA, USA) at 1:500 dilution, and developed with 2-2′azinobis (3-ethylbenzthiazoline-6-sulphonic acid) diammonium salt (ABTS; Sigma). The reaction was stopped using 0.1 mol/L citric acid and read at an optical density of 405 nm. The standard curves were made with recombinant mouse LCN2/NGAL or recombinant human LCN2/NGAL (both from R&D Systems). The detection limit for the mice assay was 50 ng/mL and for the human was 0.5 ng/mL, being the linearity of the assay for the human samples from 0.5–10 ng/ml and for the mouse from 400–800 ng/ml.

### Statistical analysis

Values are reported as mean ± SE. Statistical significance was determined using the nonparametric Mann–Whitney U test, and ANOVA analysis with differences considered significant at *p* < 0.05 (^*^) and *p* < 0.01 (^**^), except for the proportion of patients with LCN2 positive CSF which was analyzed with the chi-square test.

## Results

### EAE induction model

**Figure 1:**
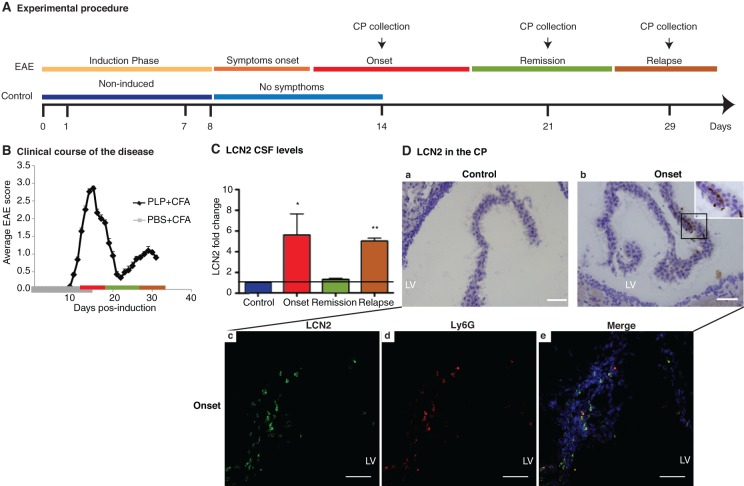
**LCN2 is increased in EAE. (A)** Immunization of SJL female mice was performed on days 0 and 7. Animals were sacrificed at the different phases of the disease and control animals at day 14. The CP transcriptome from the onset, remission, and relapse phases were compared with the CP transcriptome of non-induced animals. **(B)** Disease course of SJL mice immunized with PLP/CFA emulsion. **(C)** Augmented levels of CSF LCN2 were observed in the EAE active phases: onset and relapse. **(D)** Immunohistochemistry analysis showed that LCN2 staining is observed in the CP stroma cells but not in the CP epithelial cells (panels **a** and **b**). Staining for Ly6G identified neutrophils as the cells labeling for LCN2 in the CP stroma (panel **c**–**e**). LV, lateral ventricle. Data represent mean ± SE. Scale bars, 50 μm.

The experimental timeline is diagramed in Figure 1A. Of the animals injected with PLP peptide in CFA, 85–90% developed an acute form of EAE (grade 3–4), which peaked around days 14–19 after immunization, and spontaneously resolved 5–6 days after peaking (Figure 1B). The remission phase was followed by a relapse phase (clinical grade 1–2) around days 29–30 after immunization (Figure 1B).

### The CP transcriptome is altered during the different phases of EAE

**Figure 2:**
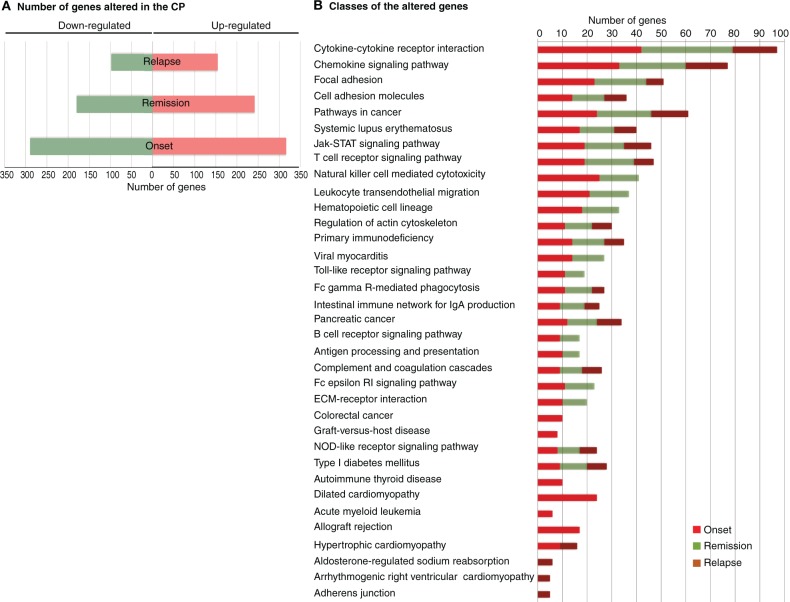
**Transcriptome signature of the EAE CP. (A)** Number of genes whose expression was found altered in the onset, remission, and relapse phases. Up-regulated genes are in red and down-regulated are in green. **(B)** Clustering of the genes whose expression was altered in the CP upon EAE induction. The number of genes altered in each pathway in the onset remission, and relapse phases are represented in red, green and brown, respectively. Microarray data are representative of one single experiment. For each time point at least 2 pools of CP were analyzed and each pool contained CP's from three animals.

**Figure 3:**
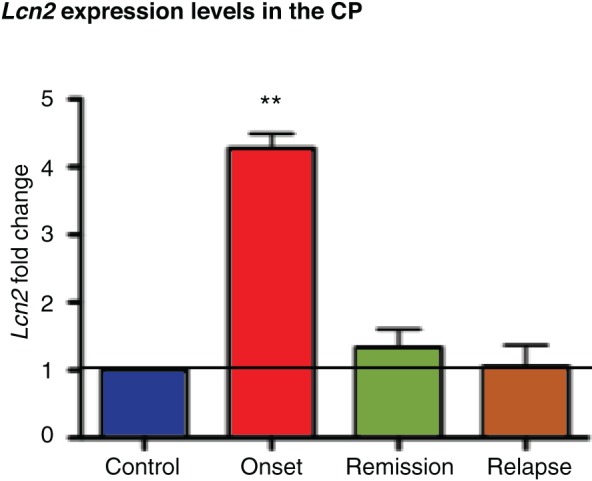
**qRT-PCR for CP *Lcn2***. *Lcn2* expression was strongly induced during the onset phase of the disease returning to control levels in the remission and relapse phases. Data are representative of one independent experiment (at least 5 pools of CP, each containing CP's from three animals). Data represent mean ± SE.

CP microarray analysis yielded several up- and down-regulated genes: 633 and 290 genes in the onset, 484 and 180 in the remission, and 309 and 96 in the relapse, respectively (Figure 2A). Gene ontology and biological pathway analysis of differentially expressed genes showed that the most altered biological pathways included chemokine signaling, focal adhesion, cell adhesion, and leukocyte transendothelial migration (Figure 2B); and genes of interest included selectins, chemokines and matrix metalloproteinases (raw data has been deposited in the GEO database, accession: GSE35363). Attending to the fold change, *Lcn2* was the most significantly up-regulated gene in the onset phase of EAE (3.1-fold change), with a reduction in expression during the remission phase (although remaining marginally up-regulated—1.33-fold change). The pattern of expression was confirmed by qRT-PCR analysis (Figure 3).

### LCN2 CSF levels correlate with the EAE active phases

We next quantified CSF LCN2 levels to determine if the CP altered gene expression profile resulted in increased protein concentration in the CSF. Interestingly, CSF LCN2 levels were comparably increased in both the EAE onset and relapse phases, while normalized in the remission phase (Figure 1C). The increased LCN2 CSF levels in the relapse phase contrasted with the lack of up-regulation in the CP gene expression in that phase, suggesting that the CSF LCN2 originates from a source other than the CP. To elucidate the LCN2 origin, immunohistochemical studies were conducted (Figure 1D). No staining was observed for LCN2 in the CP epithelial cells in any phase of the disease but rather in the CP stroma in the onset phase (Figure 1D, panels **a** and **b**). Staining in stroma was localized and restricted to infiltrating neutrophils (double staining with neutrophil marker Ly6G; Figure 1D, panels **c–e**).

### A dual source of LCN2 in the brain parenchyma

**Figure 4:**
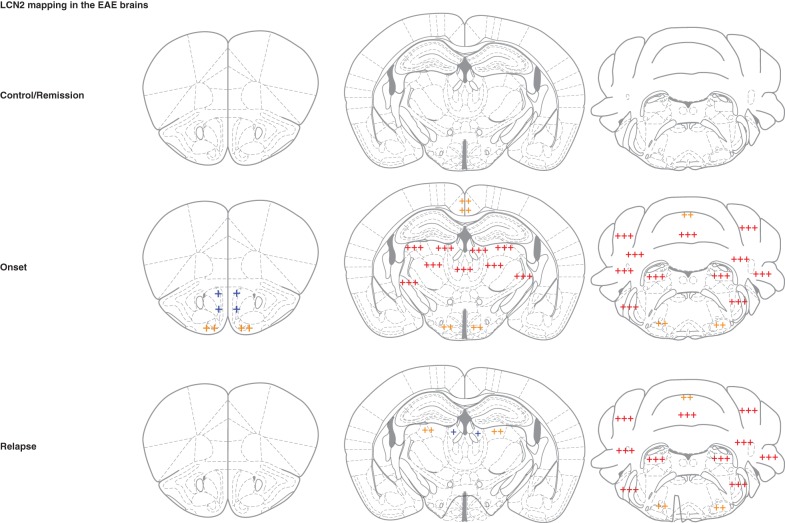
**Schematic representation of the LCN2 expression distribution in the EAE brains**. Staining was mostly observed in the olfactory bulb, periventricular regions surrounding the lateral and 3rd ventricles, cerebellum and brain stem in the active phases of EAE. The cerebellum and the brain stem were the regions more highly stained in the relapse phase. No DAB-positive staining was observed in the brain parenchyma of control animals or in the remission phase of the disease. +++, high expression; + +, medium expression; +, low expression. Data are representative of two independent experiments each containing four animals/group.

**Figure 5:**
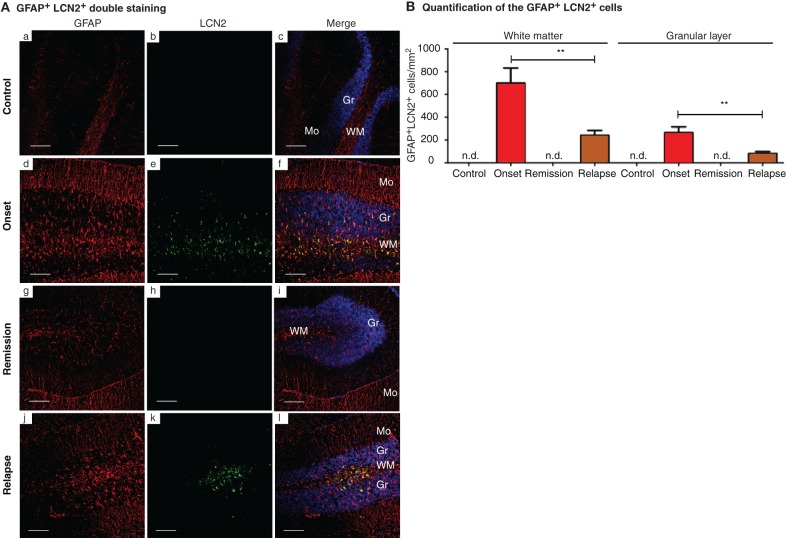
**LCN2 is produced by astrocytes. (A)** Double immunofluorescence labeling for LCN2 (green) and of GFAP for astrocytes (red) indicated double-labeled cells in the onset and in the relapse phases (panels **d**–**f** and **j**–**l**), while no staining was observed in the remission phase or in controls (panels **a**–**c** and **g**–**i**). **(B)** Localization of the double positive staining was restricted to white matter and to the granular layer of the cerebellum; in the onset and relapse phases of the disease. None or few cells were found in the cerebellum molecular layer. Mo, molecular layer; Gr, granular layer; WM, white mater; n.d., not found. Data represent mean ± SE. Scale bars, 100 μm.

To further investigate possible sources for the LCN2 present in the CSF, whole brain parenchyma immunohistochemical analysis was performed. In the onset phase, LCN2 staining was observed in the olfactory bulb, periventricular regions surrounding the lateral and 3rd ventricles, cerebellum, and brain stem (Figure 4). In contrast, in the relapse phase, the cerebellum and the brain stem were the most highly stained regions. Double immunofluorescence studies using markers for oligodendrocytes (CNPase), microglia (Iba1), neurons (NeuN) and astrocytes (GFAP), indicated that, apart from infiltrating neutrophils, the only cells producing LCN2 were astrocytes (Figure 5A). Of notice, LCN2 expression by neutrophils and astrocytes was only observed in the onset and relapse phases (Figure 5A, panels **d**–**f** and **j**–**l**), in accordance with CSF protein levels (Figure 1C).

Given that in MS patients the cerebellum is commonly affected (Calabrese et al., 2010), we further analyzed the astrocytic LCN2 regional expression in this structure. LCN2 was mainly expressed in the white matter and in the granular layer with no cells found in the molecular layer (Figure 5A). Quantitatively, the number of stained cells was higher in the onset than in the relapse phase of EAE (Figure 5B), which correlated with the animals' clinical score (Figure 1B).

### Natalizumab treatment modulates LCN2 expression

**Figure 6:**
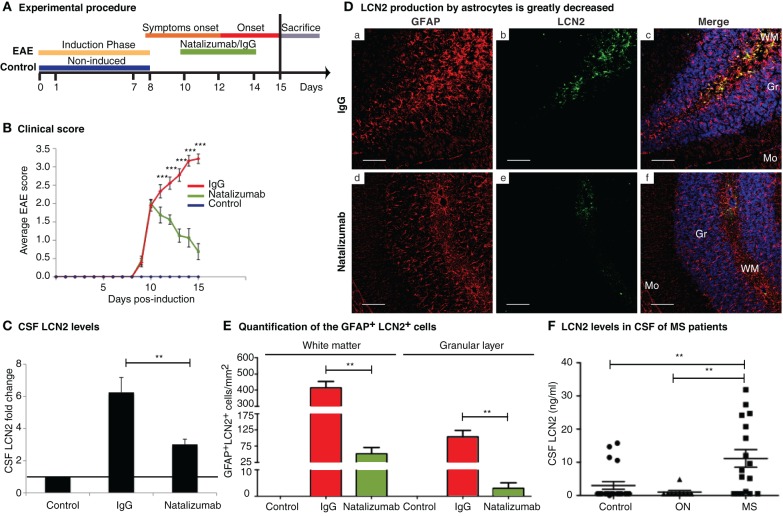
**Natalizumab abrogates the expression of LCN2 and LCN2 is increased in the CSF of MS patients. (A)** After EAE induction animals were injected intraperitoneally with natalizumab or IgG (control for the treatment) 1, 3, and 5 days after the appearance of clinical symptoms. Animals were sacrificed at the onset phase for the analysis of LCN2. **(B)** Treatment attenuated the disease clinical score. **(C)** CSF LCN2 levels were modulated and normalized by natalizumab treatment. (**D** and **E**) LCN2 astrocytic labeling was strongly reduced upon natalizumb treatment both in the white matter and in the granular layer of the cerebellum. **(F)** LCN2 was detected in 5 out of 20 control samples; 1 out of 9 ON patients and in 12 out of 20 patients with MS. CSF levels were significantly higher in MS patients when compared to the non-MS individuals. Data are representative of two independent cohorts from Portugal and Denmark. Data represent mean ± SE. CSF samples below the detection (0.5 ng/ml) were given the value of 0.5 ng/ml. Mo, molecular layer; Gr, granular layer; WM, white mater. Scale bars, 100 μm.

Natalizumab, one of the most effective MS treatments, is a humanized mouse monoclonal antibody against the integrin very late activation (VLA)-4 on leukocytes (Iaffaldano et al., 2011) that blocks leukocyte entry into the CNS. Natalizumab treatment (Figure 6A) ameliorated the clinical manifestations of the disease (clinical score 0.5, Figure 6B) and significantly decreased LCN2 CSF levels (Figure 6C); which corresponded to an abrogated expression of LCN2 in astrocytes (Figures 6D,E) and to a reduction of neutrophils infiltrating the brain parenchyma (data not shown), when compared to IgG-treated animals.

### LCN2 is increased in CSF samples from MS patients

To investigate whether the findings in the murine EAE model were also observed in humans, LCN2 CSF levels were analysed in two separate MS cohorts. Samples were divided in three clinical groups: controls (age and gender matched), patients with monosymptomatic ON and patients with clinically active MS. LCN2 was detected in 5/20 (25%) control samples, in 1/9 (11%) ON, and in 12/20 (60%) MS samples [χ^2^_(df = 5)_ = 13.98; *p* = 0.0158). Additionally, CSF LCN2 levels were significantly higher in MS patients when compared to controls and ON patients (Figure 6F).

## Discussion

Here we identify LCN2 as a novel protein whose presence in the CSF and expression in the brain parenchyma (astrocytes and infiltrating neutrophils) correlates with the active phases of the EAE mice model and is reverted to normal levels with natalizumab treatment. Additionally, LCN2 CSF levels were found significantly higher in MS patients when compared to healthy individuals or ON patients. These observations suggest that LCN2 CSF levels may be of relevance to include in the panel of MS CSF markers. In addition, we describe the CP transcriptome in the onset, relapse, and remission phases of EAE. Of notice, the most altered biological pathways were associated with leukocyte migration to the CNS, supporting the CP as a possible route of initial leucocytes entry into the brain (Vercellino et al., 2008; Reboldi et al., 2009). Several additional pathways were found altered and should be further investigated in order to understand whether specific modulators of disease onset and progression are present at the BBBs.

We have previously analyzed the CP transcriptome in the context of the interplay between the periphery and the brain in response to acute and repeated peripheral inflammation (Marques et al., 2009a,b). Interestingly, the CP response differed depending on the stimulus. Overall, the CP transcriptome changes appeared to be more pronounced in response to a transient and acute stimulation than during continuous (chronic) stimulation, in which there seemed to be an adaptation of the response. Portraying this, the highest observed fold changes we observed in the present study were in the order of 3-fold magnitude, indicating that EAE induction triggers a blunted CP response more akin to that observed upon repeated peripheral inflammation (3–4 fold) (Marques et al., 2009b) than after an acute inflammatory challenge (up to 50-fold) (Marques et al., 2009a). Furthermore, specifically for *Lcn2* (here the highest up-regulated gene), previous work showed that in the presence of an acute stimulus, changes were restricted to CP epithelial cells (Marques et al., 2008), when compared to a response by the CP stromal cells in the presence of a chronic stimulus in which no epithelial cells displayed LCN2 staining. Here, the LCN2 cell expression pattern appeared again to parallel that of the chronic model since LCN2 was found expressed by neutrophils, a stromal cell population known to extensively infiltrate the brain in the acute phase of EAE mice model and also in MS patients (Wu et al., 2010; Naegele et al., 2012), and in astrocytes, but not in CP epithelial cells. The relevance of LCN2 in EAE is further strengthened by the observation that the gene and the protein are also increased in the spinal cord, and that the severity of the symptoms are higher in *Lcn2*-null mice, as recently described (Berard et al., 2012).

Lipocalin 2 was first described as an acute phase protein involved in the innate immunity given its ability to bind bacterial siderophores (Flo et al., 2004). More recently, the identification of mammalian siderophores (Devireddy et al., 2010) suggested a novel function for LCN2 in iron homeostasis, as a protein able to deliver iron to cells by a transferin-independent mechanism (Devireddy et al., 2005). Therefore, either through its ability to modulate iron delivery or through mechanisms still to be identified, the current evidence suggests additional functions for LCN2.

Further insights into the role of LCN2 in MS can be gleaned from our novel finding revealing the expression of LCN2 in brain astrocytes of EAE mice, exclusively during the active phases of disease. Interestingly, Berard et al. (2012) have similarly described LCN2 label in spinal cord astrocytes in EAE; but not in postmortem brain tissue from patients with MS; which may be attributed to differences between humans and rodents, or to the stage of the disease. Even though the participation of astrocytes in MS has been poorly investigated, astrocytes have been shown to have an active role in axonal-associated damage during the early phases of murine EAE (Wang et al., 2005). Further understanding on whether LCN2 is a marker of activated astrocytes alone, or represents a key mediator in the astrocytes involvement in MS pathogenesis might have implications for the development of neuroprotective therapeutic strategies, which deserves further research. Also of relevance will be to investigate whether the decrease in astrocytic LCN2 during the remission phase results from apoptosis of reactive astrocytes (Lee et al., 2009) or from a decreased inflammatory response due to the lower number of infiltrating immune cells. Similarly, the lower number of astrocytes producing LCN2 upon treatment may relate with natalizumab's ability to diminish immune cell entry into the brain.

LCN2 has been recently suggested to participate in the initiation of inflammation and leukocyte migration into injured spinal cord sites (Rathore et al., 2011) and, in the brain, as a promoter of cell migration that occurs through chemokine up-regulation (Lee et al., 2011). However, the observation that EAE *Lcn2*-null mice have increased number of infiltrating immune cells in the lesions, when compared to EAE wild-type mice (Berard et al., 2012), questions the role of LCN2 in cell migration. LCN2 is also known to modulate iron availability (Richardson, 2005), which is of relevance since iron is necessary for myelinization. Importantly, iron seems to accumulate in human MS brain lesions and also in mice; in the latter, treatment with the iron chelator desferrioxamine reduces clinical and pathologic signs of EAE (Pedchenko and LeVine, 1998).

The results obtained in the murine model were translatable into humans, which corroborated the relevance of the findings. MS patients from two different cohorts presented increased CSF LCN2 levels. These observations are in line with the increased LCN2 levels in the serum of relapsing-remitting MS patients relative to healthy controls (Berard et al., 2012). Here we showed that LCN2 CSF levels of MS patients are increased in comparison with healthy controls and ON patients. These observations in humans are also in line with two separate GEO database entries on EAE/MS studies, where *Lcn2* expression appears 50% increased in post-mortem brains of MS patients (GDS2978) and in spinal tissue from EAE animals (GDS510). Altogether, these data support the inclusion of LCN2 CSF levels in the panel of inflammatory MS markers to monitor therapy and disease progression. One important question that should be addressed in the future is to evaluate whether LCN2 levels rise earlier than current diagnostic markers of the disease. CSF analyses are often performed as an aid to diagnosis of MS, especially in the early presentations, since the presence of OCB doubles the risk of developing MS after a first demyelinating event (Tintore et al., 2008). However, many patients with positive OCB might never develop the disease, particularly when the imaging studies are less informative (Tintore et al., 2008). Finding of additional biomarkers of disease, including in the CSF, is therefore of clinical relevance so to identify patients that would benefit from early treatment (Comi et al., 2000). Interestingly, five MS samples had no measurable CSF LCN2: of these, three also did not present OCBs, and four corresponded to patients with longer remission periods. In the present study we not only compared MS patients with controls, but also with ON patients. ON is often seen as the debut symptom in MS and approximately 50% of patients with ON convert into MS within 6 years (The Optic Neuritis Study Group, 2008). However, when no white matter lesions are detected, the risk of ON patients to develop MS in a 15-year period decreases, but is still higher than that of controls. The ON patients in this study were at low risk of progressing to MS due to normal MRI or to the lack of OCB. The follow-up of these individuals is crucial to determine whether the absence of LCN2 expression found in most of them is a prognostic marker for no progression into MS or, otherwise, whether the presence of LCN2 in ON individuals correlates with conversion into MS.

Here we identify LCN2 as a novel protein whose presence in the CSF and expression in the brain parenchyma is unique to the active phases in the EAE mice model. Additionally we found that the astrocytic LCN2 expression was restricted to the regions typically affected in MS patients. Since LCN2 CSF levels were also found significantly elevated in MS patients the observations support LCN2's inclusion in the panel of MS CSF markers.

### Conflict of interest statement

The authors declare that the research was conducted in the absence of any commercial or financial relationships that could be construed as a potential conflict of interest.
